# Stability and SERS signal strength of laser-generated gold, silver, and bimetallic nanoparticles at different KCl concentrations

**DOI:** 10.1016/j.heliyon.2024.e34815

**Published:** 2024-07-18

**Authors:** Vita Petrikaitė, Martynas Talaikis, Lina Mikoliūnaitė, Aikaterini-Maria Gkouzi, Romualdas Trusovas, Martynas Skapas, Gediminas Niaura, Evaldas Stankevičius

**Affiliations:** a Department of Laser Technologies, Center for Physical Sciences and Technology (FTMC), Savanoriu 231, LT-02300, Vilnius, Lithuania; b Department of Organic Chemistry, Center for Physical Sciences and Technology (FTMC), Sauletekio Ave. 3, LT-10257, Vilnius, Lithuania; c Department of Characterization of Materials Structure, Center for Physical Sciences and Technology, Sauletekio Ave. 3, LT-10257, Vilnius, Lithuania

## Abstract

Noble metal nanoparticles, specifically gold and silver, are extensively utilized in sensors, catalysts, surface-enhanced Raman scattering (SERS), and optical-electronic components due to their unique localized surface plasmon resonance (LSPR) properties. The production of these nanoparticles involves various methods, but among the environmentally friendly approaches, laser ablation stands out as it eliminates the need for toxic chemicals during purification. However, nanoparticle aggregation poses a challenge in laser ablation, necessitating the addition of extra materials that contaminate the otherwise clean process. In this study, we investigate the effectiveness of a biocompatible material, potassium chloride (KCl), in preventing particle aggregation. Although salt is known to trigger aggregation, we observed that certain concentrations of KCl can slow down this process. Over an eight-week period, we examined the aggregation rate, extinction behavior, and stability of gold, silver, and hybrid nanoparticles generated in different KCl concentrations. Extinction spectra, SEM images, SERS signal strength, and zeta potential were analyzed. Our results demonstrate that laser ablation in water and salt solutions yields nanoparticles with a spherical shape and a negative zeta potential. Importantly, we identified the optimal concentration of potassium chloride salt that maintains solution stability and SERS signal strength. Adsorbed chloride ions on silver nanoparticles were evidenced by low-frequency SERS band near 242 cm^−1^. A better understanding of the effect of KCl concentration on the properties of noble metal nanoparticles can lead to improved generation protocols and the development of tailored nanoparticle systems with enhanced stability and SERS activity.

## 1 Introduction

Gold, silver, and their alloy counterparts, such as AgAu nanoparticles, have gained significant attention due to their diverse optical, electronic, and catalytic properties, which are closely related to the localized surface plasmon resonance (LSPR) phenomenon. LSPR is a collective oscillation of conduction electrons in response to incident electromagnetic radiation, resulting in a strong electromagnetic field enhancement near the nanoparticle surface. This enhancement leads to unique optical phenomena, such as enhanced light scattering and absorption. Due to their unique properties [1,2] and wide range of applications, the synthesis and use of noble metal nanoparticles have revolutionized various fields of science and technology [3]. They are used in sensors [[3], [4], [5]], nanotechnology, catalysts [[6], [7], [8], [9]], biological applications [[10], [11], [12], [13], [14], [15]], surface-enhanced Raman scattering (SERS) [[16], [17], [18], [19], [20], [21]], drugs detection [22], labeling [23], and electronics [24,25]. The synthesis of noble metal nanoparticles has been extensively studied. Various methods have been developed, including chemical reduction [26,27], electrochemical deposition [28], sol-gel processes [29], and laser ablation [[30], [31], [32], [33], [34], [35], [36], [37], [38]]. Among these techniques, laser ablation has gained momentum as a clean and environmentally friendly method for nanoparticle generation. In laser ablation, a high-energy laser pulse is focused on a target material immersed in a liquid medium, causing rapid heating and vaporization of the target material [32,38]. The resulting vapor quickly cools and condenses, resulting in the formation of nanoparticles in the surrounding solution. An advantage of laser ablation is its ability to produce nanoparticles without the use of additional chemical reductants or stabilizers, minimizing the risk of contamination. It was demonstrated that size of nanoparticles can be controlled without employing artificial ligands in a wide range (4–400 nm) by laser post-irradiation, delayed bioconjugation, and addition of low salt concentration electrolytes [38]. In addition, laser-ablation technique can be used for synthesis of bimetallic nanoparticles (such as AuAg) directly from the bulk target without employing complex reductants [38].

However, one of the challenges associated with laser ablation, is the tendency of the nanoparticles to aggregate, which limits their stability and hinders their subsequent applications. Aggregation occurs due to the attractive van der Waals forces between nanoparticles, which can lead to irreversible agglomeration and loss of desired properties [[39], [40], [41]]. To overcome the problem of particle aggregation during laser ablation, researchers have explored the use of additives or stabilizers that contaminate the otherwise clean process. In this study, the effect of KCl concentration on the formation, stability, and surface-enhanced Raman scattering (SERS) signal strength of gold, silver, and bimetallic noble metal nanoparticles synthesized by laser ablation is investigated. SERS is a powerful analytical technique that utilizes the enhanced electromagnetic field near metal nanoparticles to amplify the Raman scattering signal of nearby molecules, enabling sensitive and selective detection of various analytes [9,17,18]. Potassium chloride (KCl), a common electrolyte and biocompatible material commonly found in biological and chemical applications, has been investigated as a potential additive to moderate particle aggregation. Based on the classical Derjaguin−Landau−Verwey−Overbeek (DLVO) theory, salt is known to promote aggregation regardless of the ion type [[42], [43], [44], [45], [46], [47], [48], [49]]. However, recent studies have shown that low concentrations of salt can instead slow down the aggregation process [50,51], according to J.P. Sylvestre et al. [50], the use of KCl and NaCl is generally analogous. It was demonstrated that nature of inorganic anion is important for stabilization of gold nanoparticles, because of specific adsorption of ions at surface of nanoparticles [52]. By carefully controlling the concentration of KCl, it may be possible to achieve a stable colloidal solution of nanoparticles without compromising their optical properties.

The above discussed works are related to our studies, but methodical analysis of different types of nanoparticles (Ag, Au, and Ag–Au) produced in different concentrations of KCl solutions in order to investigate their stability and SERS suitability has not been carried out to the best of our knowledge. In this work bulk targets of high-purity gold and silver are immersed in water solutions containing different concentrations of KCl and subjected to laser ablation with a focused Nd:YAG laser. The resulting colloidal solutions are characterized using a variety of techniques, and the ablation process is carefully controlled to ensure reproducibility. The aggregation rate and extinction behavior of the nanoparticles are monitored over a period of 8 weeks, with weekly measurements of extinction spectra and photographs taken to track any changes in the colloidal solutions. In addition, the particle morphology, SERS signal strength, and zeta potential are examined to provide a comprehensive understanding of the properties of the synthesized nanoparticles.

The main novelty of our work lies in a systematic analysis of the effect of KCl on stability and SERS performance of three types of gold and silver colloidal solutions (monometallic, monometallic mixed with different metal sequences, and alloys) prepared by laser ablation. Optical properties of all of these colloidal solutions were compared, and localized plasmon resonance peaks for each solution were identified. By studying the effect of different KCl concentrations on the nanoparticle formation process we aim to determine the threshold concentrations at which stable colloidal solutions can be obtained. These findings will contribute to the optimization of the laser ablation process and the understanding of the role of KCl as a potential stabilizing agent in the preparation of noble metal nanoparticles and expand their potential applications.

## 2 Materials and methods

### 2.1 Materials and parameters used in nanoparticle generation

**Fig. 1:**
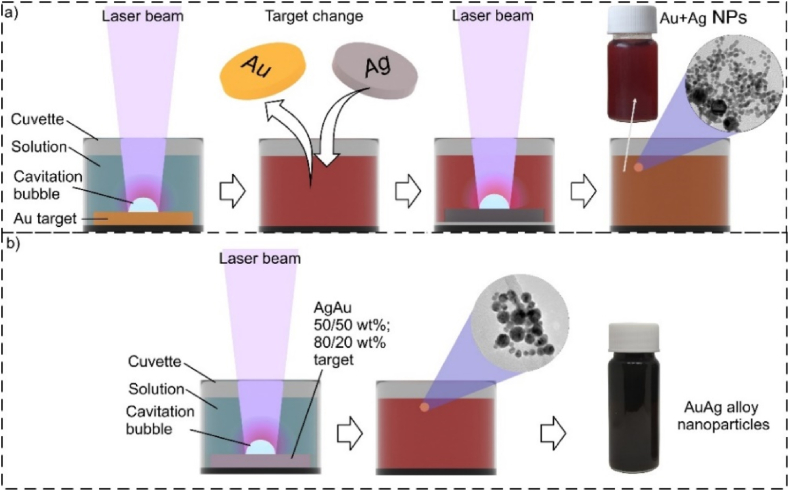
Generation schemes for nanoparticles: a) mixed monometallic and b) monometallic and alloys.

Three types of colloidal solutions were generated in this study: monometallic (Au, Ag), monometallic mixed (with different metal sequences: Au + Ag, Ag + Au), and from alloys (AgAu 50/50 wt%, AgAu 80/20 wt%), all generated under identical focusing and treatment conditions. A nanosecond first harmonic laser (1064 nm) was used with pulse duration and energy of 10 ns and 700 μJ, respectively. The energy density on the target during the laser ablation was ∼7 J/cm^2^. A scan speed of 500 mm/s and a repetition rate of 10 kHz were selected. The same area of each sample was scanned twice, one scan consisting of two 50 μm hatches with different scan angles: 0°, and 90°. Gold, silver and mixed (Au + Ag, Ag + Au) nanoparticles were generated from bulk Au and Ag targets (99.97 % in purity), alloys from (AgAu 50/50 wt%, AgAu 80/20 wt%) bulk targets (99.99 % in purity) immersed in 20 ml deionized water solutions with different KCl concentrations: 0, 0.1, 1, 2.5, 5, 10, 15, and 20 mM. The average size and concentration of all nanoparticles in the KCl solution varied from 5 nm to 43 nm and 30 mg/l to 180 mg/l, respectively (Table S1 and Table S2), depending on the salt concentration and particle material. Concentrations of Au and Ag NPs in monometallic mixed solution at different concentrations of KCl solutions are given in Table S3. The experimental setup is shown schematically in Fig. 1. Mixed solutions were generated from different bulk targets, ablating one metal target first and then the other in the colloid obtained from the former (Fig. 1a). The sequence of metal targets was varied and its influence was noted. Gold, silver, and alloy colloids were prepared by a one-step method (Fig. 1b). The total generation time and area were the same in both cases (t = 1.2 min; A = 4.34 cm^2^). During laser ablation of bulk targets, a plume of plasma is visible on the surface, the intensity of which depends on the laser energy and the focused beam [53]. Samples were ablated at the focal point. The position between the target and the lens and the thickness of the water layer above the target surface determined the position of the focal point. This was due to the focal plane shifting caused by water's optical refraction.

### 2.2 Characterization of the samples

Extinction spectra and photographs of the formed colloidal solutions were recorded weekly for a total of eight weeks. The UV–Vis absorption spectra were collected in solution using a quartz cuvette (light path: 10 mm). The UV–Vis extinction spectrum were recorded with an *Essentoptics Photon RT UV-VIS-MWIR* spectrophotometer. Spectra were measured in the 300–750 nm wavelength range with a 4 nm data acquisition step at an 8-degree angle of incidence. A spot size of 2 × 5 mm^2^ was used during the measurements.

Laser microelectrophoresis was used to measure the zeta potential with a *Malvern Zetasizer Nano ZS*. There were 10 measurements taken and averaged.

For nanoparticle SERS characterization, a 4-mercaptobenzoic acid (4-MBA) self-assembled monolayer (SAM) was first formed. A 150 nm-thick Au layer was deposited on microscopic glass slides using a PVD75 magnetron sputtering system (Kurt J. Lesker Co., US). Immediately after that, the slides were transferred to 1 mM 4-MBA ethanol incubation solution for 2 h, then rinsed and dried under the N_2_ stream. After that, 10 μl of the selected nanoparticle solutions were dried on the Au slides. SERS measurements were carried out by using an *inVia* Raman spectrometer (Renishaw, Wotton-under Edge, UK) equipped with a thermoelectrically cooled (−70 °C) CCD camera and He–Ne laser producing 633 nm wavelength radiation and 1800 lines/mm grating. Each nanoparticle droplet was sampled at 50 locations with 10 s accumulation time. 3–5 droplets of the same nanoparticle composition were analyzed, and the results were averaged. For SERS measurements of adenine (≥99%; Sigma-Aldrich, St. Louis, MO, USA), selected nanoparticle solutions (10 μl) were dried on a steel substrate, and then 10 μl 10^−5^ M of adenine were dried on top. Measurements were carried out at 15 different places of the sample. All measurements were conducted at 45 μW laser power focused on a sample surface of approximately 1 μm in diameter using a 50 × /0.75 NA objective lens (Leica).

## 3 Results and discussion

### 3.1 Properties of gold and silver nanoparticles from a bulk target

**Fig. 2:**
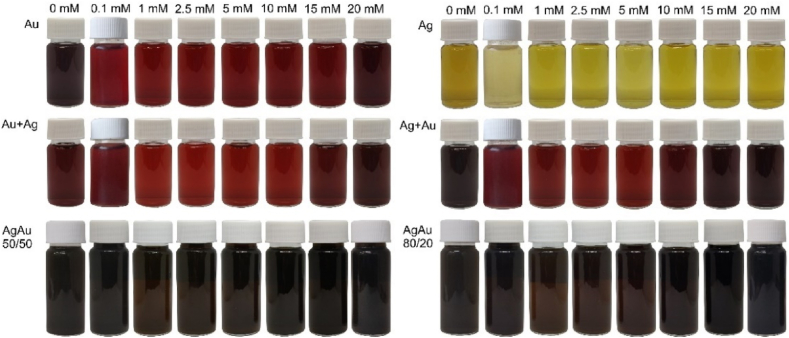
Photographs of the Au, Ag, Au + Ag, and Ag + Au and alloy AgAu 50/50 wt% and 80/20 wt% colloidal solutions on day one. The molar concentration of KCl in an aqueous solution from 0 to 20 mM is indicated above.

Firstly, the post-generation colors of the colloid and their corresponding extinction spectra are analyzed, which are good indicators of nanoparticle size and stability due to the size-dependent properties of surface plasmon resonance [30,54,55]. Samples assayed in this work from monometallic gold, silver, and alloy targets on the first day of formation are shown in Fig. 2. They were poured into 20 ml vials and then placed according to the target and KCl concentration indicated above the vials.

**Fig. 3:**
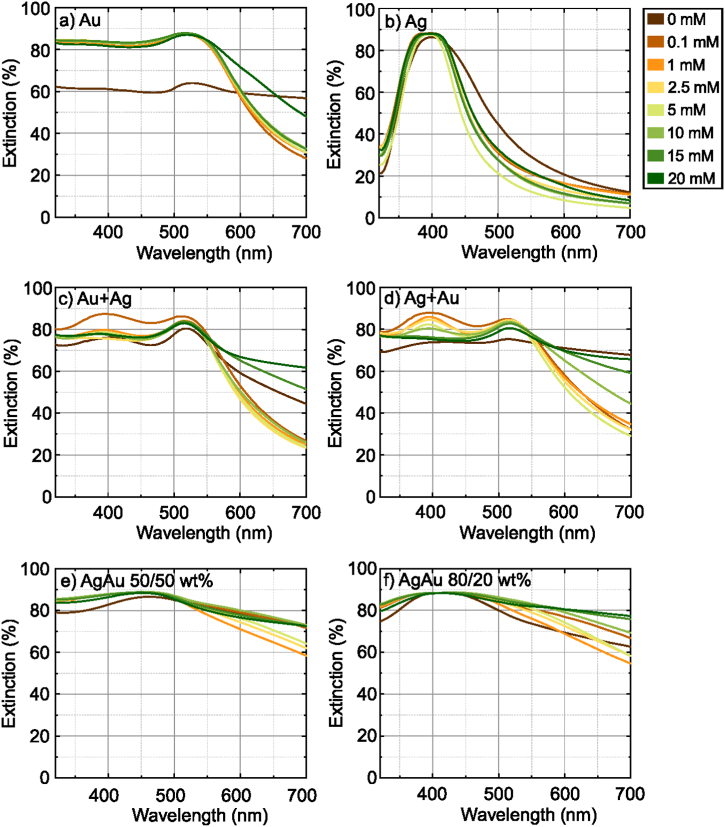
First-day extinction spectra of gold (a), silver (b), Au + Ag (c), Ag + Au (d) and alloy AgAu 50/50 wt % (e), AgAu 80/20 wt% (f) nanoparticles generated in water (brown spectrum) and KCl solutions of different concentrations. (For interpretation of the references to color in this figure legend, the reader is referred to the Web version of this article.)

**Fig. 4:**
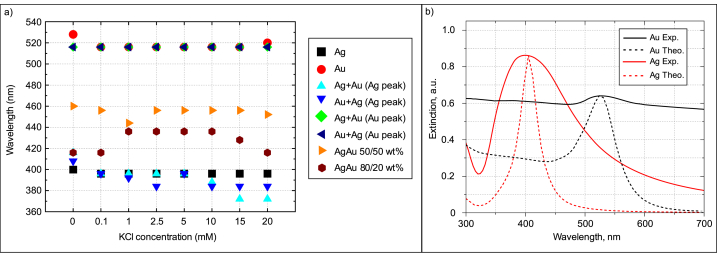
a) Localized plasmon resonance peaks. The double peaks of the mixed gold-silver nanoparticles are separated by the metal corresponding to that peak; b) Theoretical (dashed lines) extinction spectra of gold and silver nanoparticles in water with sizes 25 nm and 33 nm, respectively. The measured extinction spectra of gold and silver nanoparticles are shown in solid lines. (For interpretation of the references to color in this figure legend, the reader is referred to the Web version of this article.)

The UV–Vis spectra of the samples from Fig. 2 on the first day of generation are shown in Fig. 3a–f. The first row in Fig. 2 shows colloidal samples of pure gold and silver targets. Colloidal gold exhibits deep red and silver–yellow hues due to localized surface plasmon resonance of the nanoparticles, this frequency is in the visible region which causes their absorption and scattering to differ from the properties of the macromaterial [9,56]. The gold and silver nanoparticles exhibited localized surface plasmon resonance peaks at ∼516–528 nm (Figs. 3a) and 396–400 nm (Fig. 3b), respectively, in agreement with previous work [32]. The second row of Fig. 2 shows mixed-metal colloidal samples generated from the same targets as in the above row, except that the first column shows samples generated from a gold target for half the time and a silver target for the other half. In the second column, the order of sample generation is reversed. These mixed samples show a similar red color as gold, due to uneven mixing with silver nanoparticles. As we have investigated in our previous work, silver has a higher ablation threshold than gold [32], so ablating the same area in the same amount of time removes less volume of the material, therefore the hybrid samples are more influenced by the gold particles and thus appear redder in color. The appearance of the two surface plasmon bands associated with both Au and Ag nanoparticles represents the formation of phase-separated Au and Ag nanoparticles [52]. For these colloids, the peak corresponding to the gold nanoparticles peaks at a uniform wavelength of 516 nm, while silver ranges from 372 nm to 415 nm (Fig. 3c and d). The same principle as for monometallic targets has also been applied to silver-gold alloys (third row in Fig. 2), where colloids in the first column are generated from AgAu 50/50 wt% target and the second column to the right from AgAu 80/20 wt% target. The conditions were the same, but as can be seen from the photographs of the samples, the result is different. Visually we see a dark brown color, although the concentration is not higher than that obtained from monometallic targets. In the case of the alloys, a single peak is observed; for the AgAu 50/50 wt% samples at 443–459 nm range (Fig. 3e) and for the AgAu 80/20 wt% at 415–436 nm range (Fig. 3f). The position of this peak is consistent with the expected linear relationship between the position of the LSPR peak and the composition of the bimetallic nanoparticles [57,58]. The samples for each metal are arranged from left to right in ascending order of the concentration of KCl in water from 0 to 20 mM (in Fig. 2). The exact plasmon bands are shown in Fig. 4a.

**Fig. 5:**
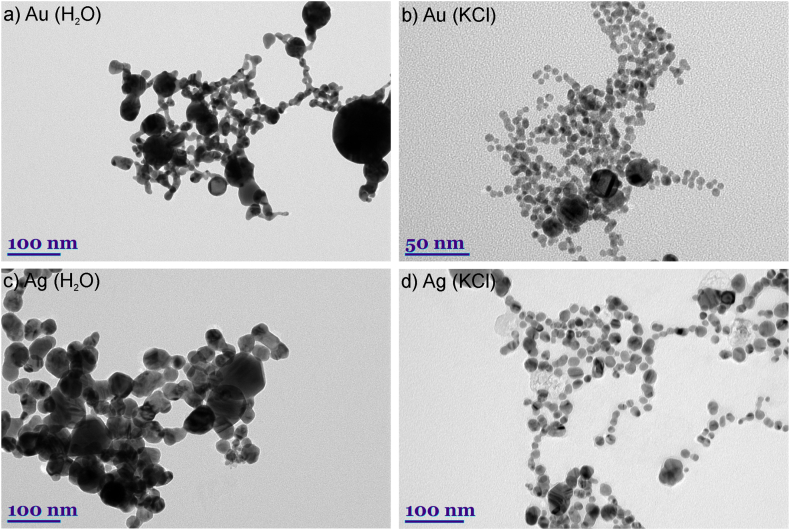
TEM images of gold (a–b) and silver (c–d) nanoparticles. Images: a) Au NP generated in water; b) Au NP generated in KCl solution (5 mM); c) Ag NP generated in water; d) Ag NP generated in KCl solution (5 mM). (For interpretation of the references to color in this figure legend, the reader is referred to the Web version of this article.)

The position, shape, and size of the LSPR peak depends not only on nanoparticle size and geometry but also on the surrounding environment [59,60]. The spectra of the particles produced in distilled water (Fig. 3, spectra in brown color) are slightly different from those produced in salt solution - the concentration is lower, which may be due to the larger particle sizes produced, judging by the color (Fig. 2), which slowly disperse in water and absorb part of the energy of the next laser pulse. It is worth noting that nanoparticles produced in distilled water by laser ablation are typically larger in size and dispersion. This behavior can be attributed to the agglomeration of nanoclusters after ablation and the ejection of large target fragments [31,53,54]. As can be seen in the spectrum (Fig. 3, spectra in brown color) and in the graph (Figs. 4a and 0 mM KCl), the peaks of gold and silver particles generated in distilled water are more red-shifted in the spectrum (528 nm) compared to those generated in salt (516 nm). The reduction in the NP size of highly dilute electrolytes (<1 mM) is directly related to the surface area of nanoparticles (NP), which can be electrostatically stabilized by free anions. Chaotropic anion accumulation in NP nanoenvironments not only affects colloid stability but also disrupts ligand-free NP growth mechanisms. Therefore, increasing ionic strength in the micromolar concentration regime significantly reduces the size and size distribution of NPs [48]. These claims are supported by TEM images of gold and silver nanoparticles generated in water and 5 mM KCl solution (Fig. 5). The TEM images were analyzed with *ImageJ* software to find the average diameter of the particles. From the images and the estimation of the average size, we can see that the nanoparticles generated in distilled water (24.4 ± 18 nm for Au and 33 ± 14 nm for Ag) differ from those generated in the salt solution (7.5 ± 5 nm for Au and 17.8 ± 9 nm for Ag) not only of their larger size but also of the way they form: in water, the particles form interconnected and branched structures, whereas in salt solution the particles remain single and relatively regular. This data indicates that small amounts of salt help to maintain the distance between the nanoparticles and inhibit the aggregation process. However, the gold nanoparticles on the left edge, generated in distilled water, have the same dark color as those generated in the high-concentration (20 mM) solution (Fig. 2), sharing a similarly shifted peak towards longer wavelengths. Using low concentrations (1–5 mM) of KCl salt, we can see that the color is much clearer, lighter, and brighter compared to nanoparticles in distilled water, while from 10 mM onwards the color of the samples darkens. This darkness and opacity are due to the larger particle size and the formation of aglomerates [42,61].

Another difference is the larger width and lower intensity of the extinction peak due to the non-uniformity of the size of the generated particles (Fig. 4b). The estimated extinction spectra (dashed lines in Fig. 4b) for gold and silver nanoparticles with sizes 25 nm and 33 nm have narrower and higher amplitude peaks compared to the measured spectra (solid lines in Fig. 4b). This result demonstrates that the size and shape of the resulting nanoparticles were not uniformly distributed, as evidenced by the TEM images (Fig. 5a–c). The spectra of monometallic particles generated in salt solutions of different concentrations (Fig. 3a and b) show little difference between the peaks, only Au nanoparticles (Fig. 3a) generated in 20 mM KCl can be distinguished; the spectrum is notable by the width of the peak due to absorption and scattering along the visible spectrum due to the range of nanoparticle size. The spectra of solutions generated from different monometallic targets differ more distinctly. As mentioned in the experimental section, bimetallic colloids were generated by ablating the monometallic bulk targets one after the other (Fig. 1a), but during this process, no alloy nanoparticles were formed, as evidenced by the two peaks in the spectra characteristic of silver and gold nanoparticles (Fig. 3c and d). The dependence of the extinction peak on the salt concentration is evident. Peaks are sequenced according to salt concentration, from particles generated at the lowest salt concentration (0.1 mM) at the top to particles at the highest concentration (20 mM) at the bottom. This pattern may indicate the onset of aggregation in the sample. High electrolyte concentrations reduce stability, particles stick together to form aglomerates and thus reduce the number of particles that have a localized surface resonance in solution, leading to a decrease in peak extinction [42]. Another observation can be made from Fig. 3c and d is that at higher salt concentrations, the peak of the silver nanoparticles in the mixed samples decreases, and from 15 mM onwards it completely flattens out. The decrease in extinction is due to the electrolyte concentration, which promotes faster oxidation, causing the silver to lose its plasmonic properties and aggregate [50,62].

The colloidal solutions of the alloys at different KCl concentrations show little variation, all having a dark brown color (Fig. 2e and f). Samples of AgAu 50/50 wt% (Fig. 4a, orange triangles) in distilled water (0 mM KCl) also show larger particle sizes compared to nanoparticles generated in salt solutions (444–456 nm), based on spectral absorption and scattering at redder wavelengths (460 nm). The blue-shifted spectrum belongs to particles generated at a concentration of 1 mM. In contrast, AgAu 80/20 wt% (Fig. 4a, brown hexagons) does not share this trend, the redder shifted spectrum is due to particles generated in salt (1–10 mM) with a peak at 436 nm. The other peaks (0, 0.1, 20 mM) are at 416 nm with the exception of 15 mM (428 nm).

**Fig. 6:**
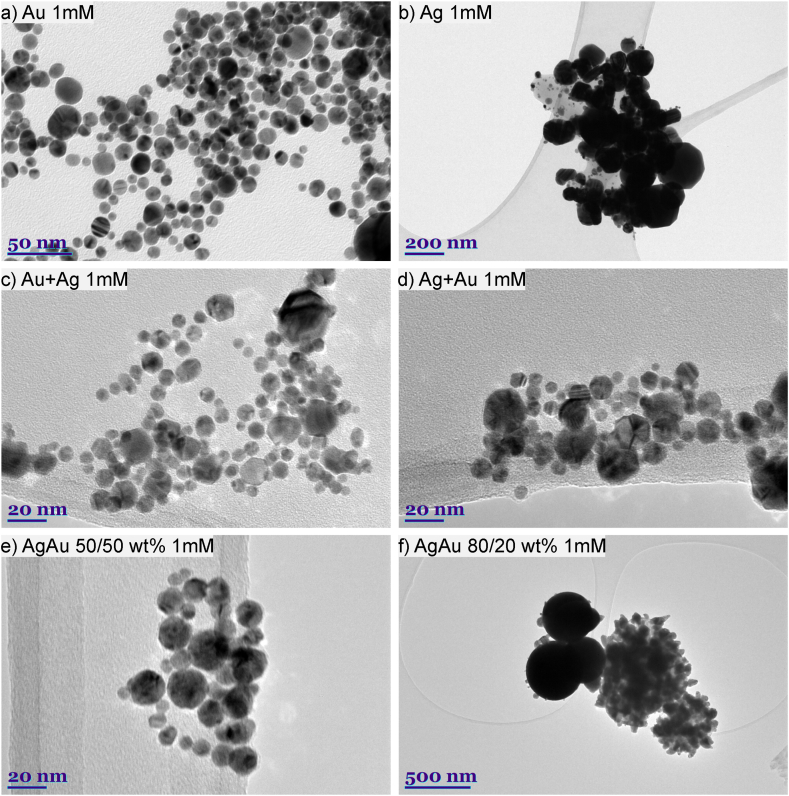
TEM images of all type of nanoparticles generated in 1 mM KCl concentration solution. Images: a) Au NP; b) Ag NP; c) Au + Ag NP; d) Ag + Au; e) AgAu 50/50 wt%; f) AgAu 80/20 wt%,.

A comparison of the morphology of the nanoparticles generated at 1 mM KCl concentration for all the targets used is shown in Fig. 6. The results obtained from TEM images showed that laser ablation of the target in saline solution produces spherical nanoparticles. The images were taken after 8 weeks, so the particles formed from silver and alloy with 80 wt% silver have a larger particle size and size dispersion as aggregates were recorded in the image.

### 3.2 Stability of colloidal solutions

Stability of the colloidal solutions was identified visually by taking weekly photographs for a total of eight weeks. Additionally, extinction spectra were measured to assess the optical properties of the nanoparticles over time. The particles were stored at room temperature, in a bright place, without additional stabilizers or protection. Zeta potential of the stable colloidal solutions was measured after 8 weeks (shown in Table S4).

**Fig. 7:**
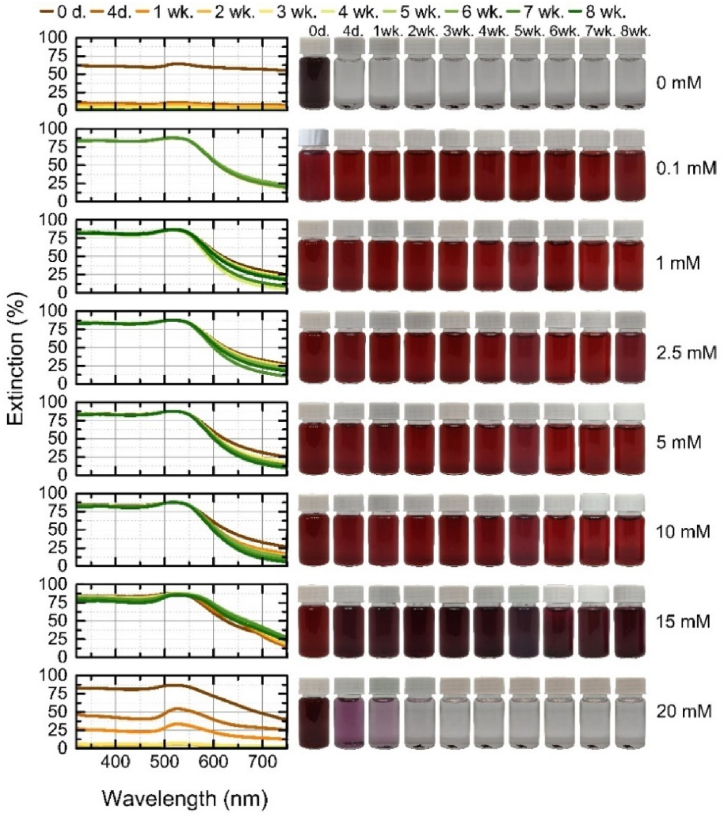
Stability of gold nanoparticles during 8 weeks: on the left – the extinction spectra of each colloidal solution at a given time; on the right – the photographs of colloidal solution at a given time. (For interpretation of the references to color in this figure legend, the reader is referred to the Web version of this article.)

The extinction spectra of monometallic gold nanoparticles over 8 weeks are shown in Fig. 7, along with the corresponding pictures of colloids. The gold nanoparticles generated in distilled water aggregated after only 4 days, with only a single agglomerate visible in subsequent vials. Nanoparticles are considered to have aggregated when they can no longer be shaken out and returned to their former state. This aggregation time is consistent with a previous study using different pulse energies [32]. The aggregation of gold nanoparticles in water is mainly decided by the electrostatic interactions of oppositely-charged nanoparticles and attractive van der Waals forces. The gold particles generated at the highest concentration of KCl (20 mM), which showed extinction at shorter wavelengths compared to the solution without electrolyte, fully aggregated within 2 weeks. In this case, a high concentration of potassium ions leads to an excess of ions in the solution, reduced thickness of the electrostatic double layer, and screening of the electrostatic charges. Different ions bond to nanoparticle surfaces with different affinities, which has a great influence on the surface charge density of the nanoparticles, and hence their stability due to electrostatic repulsion, according to the Hofmeister or lyotropic effect [47]. All these effects neutralize the charges on the colloidal particles or reduce the repulsive forces between the particles, leading to their aggregation [51,63,64]. The last measurement of extinction spectra of the highest concentration of KCl with the LSPR peak before aggregation shows that the LSPR is red-shifted from 520 nm to 528 nm and the color of the solution took on a bluer hue, which is related to the higher concentration of salt in solution [46,50]. At lower concentrations (0.1–15 mM), the gold nanoparticles remained stable for 8 weeks. At a concentration of 0.1 mM, the spectrum remained constant over the whole time period due to the high negative zeta potential (ζ = −53 mV). At 1–10 mM the peak showed only slight changes, its position remained the same (516 nm) but narrowed slightly. This narrowing was due to the sedimentation of larger fragments. Particles generated at 2.5 mM KCl had the highest negative potential, with a ζ-potential of −55 mV, while those generated at 5 mM had the lowest (−19 mV). A greater change in the spectrum is seen with 15 mM KCl solutions, with the color darkening to a bluer shade on day 4 due to a change in extinction that shifted the peak from 516 nm to 520 nm and then from the first week to 524 nm. The shift in extinction is due to the interaction between the nanoparticles, whereby the nanoparticles clumped together into larger derivatives with absorption corresponding to the higher wavelength, and maintained that state for the rest of the weeks. This result is due to the contribution of free electrons to the dielectric function, which changes with particle size. The frequency required to initiate plasmon oscillations decreases. This is caused by the electromagnetic decay resulting from the depolarization of the light field across the surface of the particle, which weakens the Coulombic restoring force acting on the electron cloud [65].

Monometallic silver nanoparticles in pure water (without potassium salt), in contrast to gold nanoparticles, were stable for the entire studied period. This is consistent with our study of pulse energy dependence, in which bulk silver generated by higher energy pulses was rather stable over five weeks [32]. As the energy used in this study was twice as high as in the previous study [32], the stability of the generated silver nanoparticles was further improved. Stability of Ag nanoparticles in pure water is associated with a relatively high surface energy of silver nanoparticles, leading to the formation of a hydration shell around the nanoparticles due to the adsorption of water molecules on the surface of the nanoparticles. This hydration shell, together with other factors such as electrostatic repulsion, dynamic equilibrium, and surface oxidation, contributes to the stability of Ag nanoparticles in pure water and helps prevent their aggregation.

**Fig. 8:**
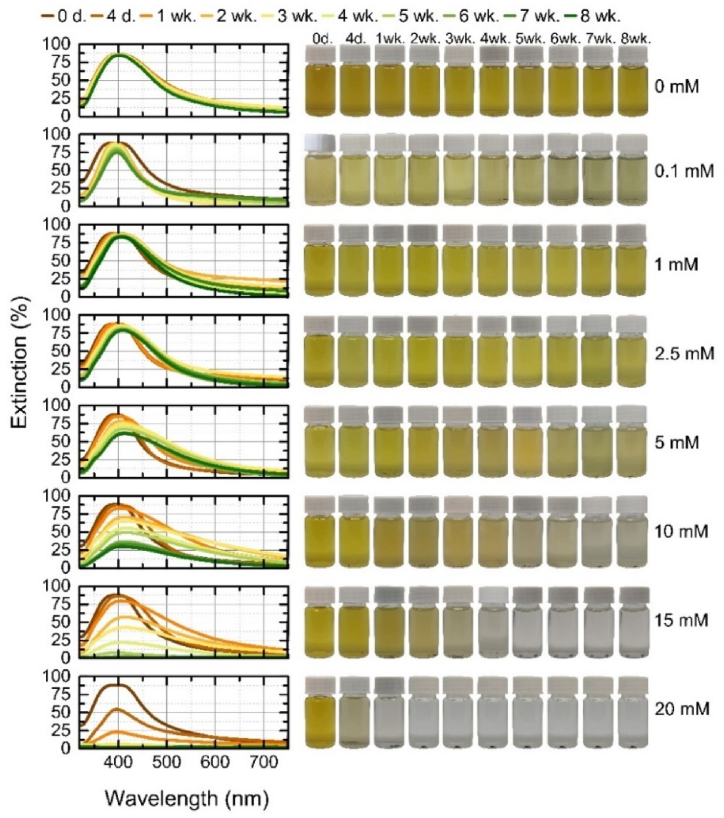
Stability of silver nanoparticles during 8 weeks: on the left – the extinction spectra of each colloidal solution at a given time; on the right – the photographs of colloidal solution at a given time.

The Ag nanoparticles generated in the salt solutions were not stable and showed a more pronounced change in spectra over 8 weeks than the gold nanoparticles (Fig. 8). The reduced stabilization efficiency of Ag nanoparticles is related to the oxidation process on the surface. Compared to gold nanoparticles, silver nanoparticles are more prone to oxidation [66]. A higher salt concentration of KCl might promote the oxidation of silver due to ion mobility [67]. As a result, the oxidized Ag surface hinders and competes with the adsorption of chloride anions, leading to a reduction in stabilization efficiency.

The process of aggregation in nanoparticles induces coupling of their plasma modes, leading to noticeable changes in the optical spectrum. Specifically, this coupling causes a red-shift, resulting in a shift towards longer wavelengths, and a broadening of the longitudinal plasma resonance [42]. At a concentration of 0.1 mM (ζ = −48 mV), the spectrum narrowed visibly from day 4 onwards, with decreasing extinction as large aggregates of finer and larger particles formed and settled on the bottom (visible in the pictures of the vials), with a red-shift only visible at week eight, when the peak shifted by 4 nm from 396 nm (400 nm). More pronounced shifts are observed at higher concentrations: 1 mM (with highest negative ζ = −52.5 mV) shifted from 396 nm by 8 nm (404 nm) in the first week and remained at 407 nm from the seventh week; 2.5 mM at 396 nm also shifted by 8 nm (404 nm) in the second week and broadened due to the formation of aggregates, and from the third week onwards it was 408 nm. At 5 mM at 396 nm peak, a shift is seen in the first week (400 nm) and a broadening of the spectrum has started, in the second week the peak is at 408 nm and the width of the spectrum has settled but the extinction further decreases, in the fourth week the peak settles at 412 nm with a further decrease in extinction. With 10 mM KCl, a more dramatic decrease in extinction and a shift from 396 nm to 416 nm was observed, with a shift of 4 nm and a broadening of the spectrum on day four and a week later, a shift of 8 nm on week two, and a shift of 416 nm on week three until week five (inclusively), when the color of the solution faded due to the aggregation of plasmonic particles, and the peaks of the spectra started to shift back towards shorter wavelengths (blue shift) but did not fully aggregate. The particles generated in the 15 mM and 20 mM solutions aggregated completely, 15 mM red-shifted from 396 nm to 416 nm in 2 weeks, and with a strong decrease in particle concentration by week four, a blue shift to 408 nm began; the 20 mM concentration aggregated completely by week 1 with no significant peak variations. Due to the electron density at the particle surface, the blue-shift can be attributed to the greater electrostatic repulsion between particles. The free electrons that are adsorbed on the particle will be confined to a smaller volume, resulting in a higher density of free electrons and thus a higher frequency of the LSPR [10,68]. The addition of potassium salt increases the ionic strength of the solution, increasing the attractive van der Waals forces between nanoparticles, leading to the aggregation of the nanoparticles.

**Fig. 9:**
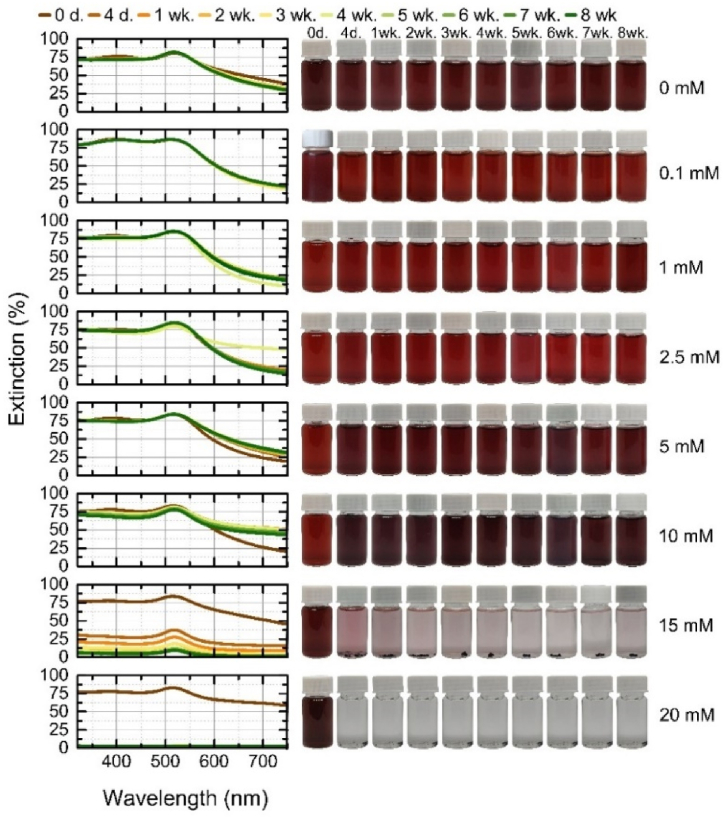
Stability of mixed Au + Ag nanoparticles during 8 weeks: on the left – the extinction spectra of each colloidal solution at a given time; on the right – the photographs of colloidal solution at a given time.

**Fig. 10:**
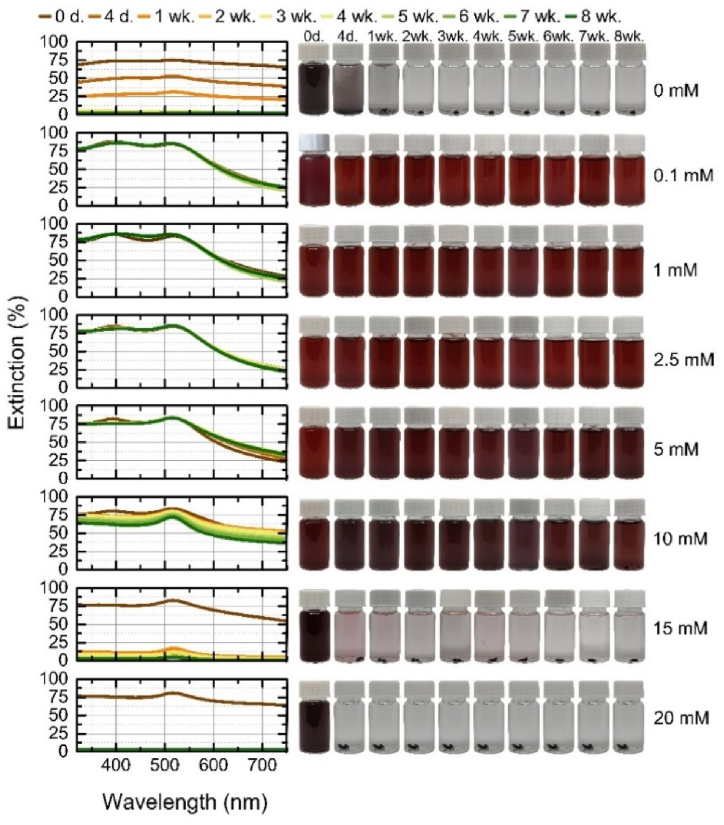
Stability of mixed Ag + Au nanoparticles during 8 weeks: on the left – the extinction spectra of each colloidal solution at a given time; on the right – the photographs of colloidal solution at a given time.

The mixed colloids were generated by ablating gold and silver bulk targets in a colloidal solution of Au or Ag in different order (Fig. 1a). The mixed colloidal solution generated by the ablation of silver target in gold nanoparticles solution is named Au + Ag (Fig. 9). Another mixed colloidal solution generated by the ablation of gold target in silver nanoparticles solution is named Ag + Au (Fig. 10). Both mixed colloidal solutions were ablated the same time period. The resulting solutions show little visual difference in color, only the lighter shade of the Au + Ag (Fig. 9) samples can be distinguished, which can be explained by the fact that the gold particles were generated first and further scanned by ablating the silver, thus by photothermal heating reducing their size and making the solution appear more transparent [30,69,70]. Furthermore, the ablation of the silver target in a KCl-free gold colloidal solution creates a stable mixed Au + Ag solution, there was no aggregation for the whole 8 weeks, in contrast to Ag + Au which aggregated in distilled water after the first week. This means that the unstable gold colloidal solution is stabilized by “freshly” ablated silver nanoparticles, creating a long-term stable colloidal solution. Conversely, a stable silver solution is destabilized by the influence of “freshly” ablated gold nanoparticles.

Zeta potential measurements at concentrations of 0–10 mM, with Ag + Au potentials ranging from −16 mV to −52 mV and Au + Ag from −29 mV to −56 mV, with the lowest negative potentials at 10 mM and the highest at 1 mM (Table S4). A high negative zeta potential of the mixed colloidal solutions indicates a high surface charge density on particles and it correlates with the high stability of the solutions. Moreover, Au + Ag in 15 mM KCl solutions held up better, and although most particles aggregated during the period, the peak was visible until the final week. In the 20 mM KCl solutions, as with the Au and Ag colloids, the particles started to aggregate on the first day. The mixtures exhibit the properties of gold and silver nanoparticles discussed earlier. In both mixtures, at all KCl concentrations, the gold nanoparticles showed stable peaks, except at higher concentrations, where the gold peak in the Ag + Au 15 mM KCl mixtures was red-shifted. The silver peak (∼400 nm) in Fig. 9 is not very evident at all concentrations of KCl. This peak is more clearly visible in the first-day extinction spectra of stable Ag + Au mixture at 1 mM–10 mM (Fig. 10). Two distinct peaks in extinction spectra indicate the presence of individual gold and silver nanoparticles in the mixture. The disappearance of a silver peak over time is related to the silver oxidation.

**Fig. 11:**
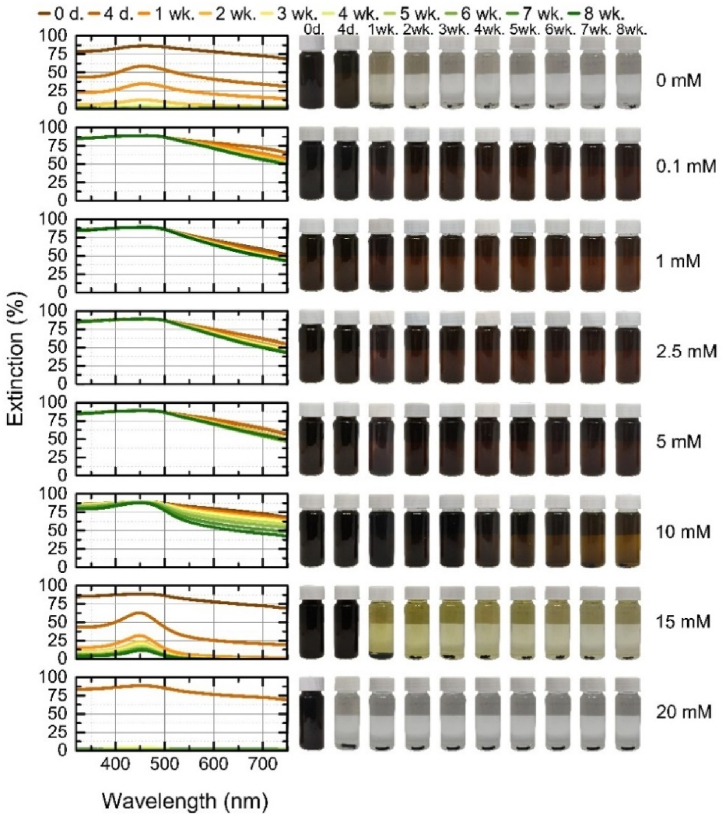
Stability of alloy AgAu 50/50 wt% nanoparticles during 8 weeks: on the left – the extinction spectra of each colloidal solution at a given time; on the right – the photographs of colloidal solution at a given time.

**Fig. 12:**
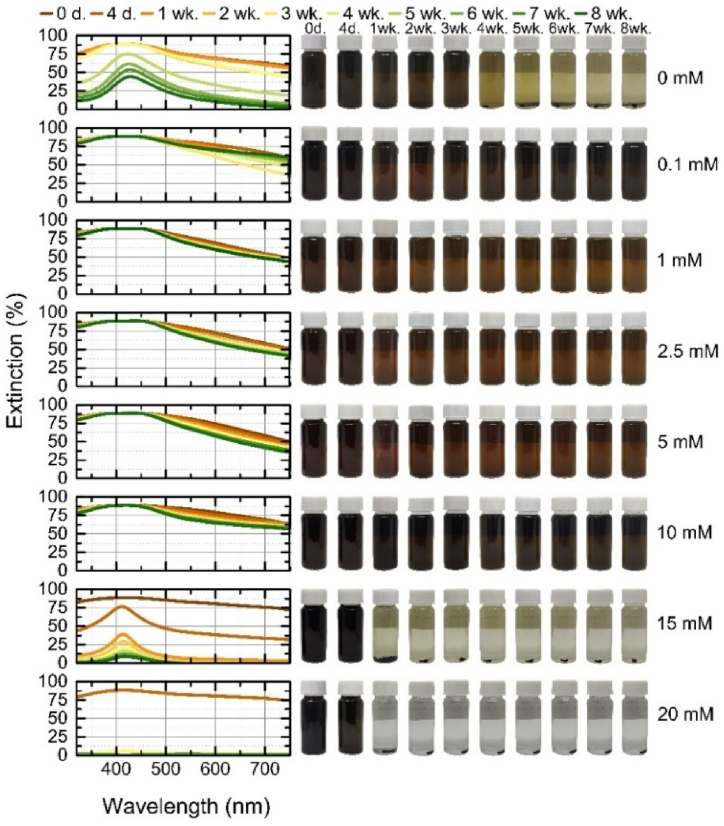
Stability of alloy AgAu 80/20 wt% nanoparticles during 8 weeks: on the left – the extinction spectra of each colloidal solution at a given time; on the right – the photographs of colloidal solution at a given time.

The stability assessment of AgAu alloys with 50/50 wt% and 80/20 wt% is demonstrated in Fig. 11, Fig. 12. As observed on the first day (Fig. 3e and f), the peaks of the alloys are very broad and of high extinction due to the high concentration and dispersion of particle sizes. It can be seen that the stability of the particles generated in distilled water varied according to the silver content of the alloy, the nanoparticles of the alloys with the higher silver content persisted for the whole time period, which was observed for the hybrid particles in the previous studies [32,38]. At concentrations of 0.1–5 mM KCl and 10 mM AgAu 80/20 wt% target nanoparticles, the spectra showed little change and remained stable over time, without significant changes. In other concentrations, a narrowing of the LSPR peak is observed, due to the sedimentation of large particles or fragments, this narrowing is more pronounced in the AgAu 50/50 wt% particles generated at 10 mM KCl. The same narrowing, accompanied by a decrease in extinction, occurs at a concentration of 15 mM and in distilled water without added salt. Due to the broad geometry of the spectrum, it is difficult to see the red-shift at low concentrations of the electrolyte, while in distilled water, the narrowing results in a shift of AgAu 80/20 wt% from 416 nm to 424 nm in the second week and 428 nm in the sixth week. The zeta potentials of the alloys at concentrations of 0.1–10 mM were as follows: the AgAu 50/50 wt% target nanoparticles ranged from −44 mV to −58 mV, with the lowest negative potential at 10 mM and the highest negative potential at 2.5 mM, while the AgAu 80/20 wt% target nanoparticles showed a negative potential ranging from −44 mV to −59 mV, with a lowest negative potential at 0.1 mM and the highest at 5 mM. In contrast to the monometallic targets, where the zeta potential increases with decreasing concentration, the potentials of the alloys showed no trend with concentration.

The stability assessment of AgAu alloys with 50/50 wt% and 80/20 wt% is demonstrated in Fig. 11, Fig. 12. As observed on the first day (Fig. 3e and f), the peaks of the alloys are very broad and of high extinction due to the high concentration and dispersion of particle sizes. It can be seen that the stability of the particles generated in distilled water varied according to the silver content of the alloy, the nanoparticles of the alloys with the higher silver content persisted for the whole time period, which was observed for the hybrid particles in the previous studies [32,38]. At concentrations of 0.1–5 mM KCl and 10 mM AgAu 80/20 wt% target nanoparticles, the spectra showed little change and remained stable over time, without significant changes. In other concentrations, a narrowing of the LSPR peak is observed, due to the sedimentation of large particles or fragments, this narrowing is more pronounced in the AgAu 50/50 wt% particles generated at 10 mM KCl. The same narrowing, accompanied by a decrease in extinction, occurs at a concentration of 15 mM and in distilled water without added salt. Due to the broad geometry of the spectrum, it is difficult to see the red-shift at low concentrations of the electrolyte, while in distilled water, the narrowing results in a shift of AgAu 80/20 wt% from 416 nm to 424 nm in the second week and 428 nm in the sixth week. The zeta potentials of the alloys at concentrations of 0.1–10 mM were as follows: the AgAu 50/50 wt% target nanoparticles ranged from −44 mV to −58 mV, with the lowest negative potential at 10 mM and the highest negative potential at 2.5 mM, while the AgAu 80/20 wt% target nanoparticles showed a negative potential ranging from −44 mV to −59 mV, with a lowest negative potential at 0.1 mM and the highest at 5 mM. In contrast to the monometallic targets, where the zeta potential increases with decreasing concentration, the potentials of the alloys showed no trend with concentration.

The effect of potassium chloride on the size and stability of various nanoparticles prepared by laser ablation is related mainly to the specific adsorption of chloride ions on Ag and Au surfaces [71,72]. The metal-chloride bonding strength is different comparing silver and gold surfaces. Higher bond covalency and higher chloride-surface charge transfer were observed by the SERS technique in the case of gold surface [72]. The surface properties of nanoparticles in the presence of adsorbed chloride ions depend on the surface coverage of ions [73]. Local changes in the work function of silver nanoparticles and the formation of specific reduced adsorption sites were detected spectroscopically at chloride coverages higher than 0.6 [73].

In summary, a small quantity (less than 10 mM) of potassium chloride in colloidal gold and silver solutions enhances their stability. This finding holds great importance in various fields, including biotechnology, electronics, catalysis, materials science, and Raman spectroscopy.

### 3.3 SERS measurements

**Fig. 13:**
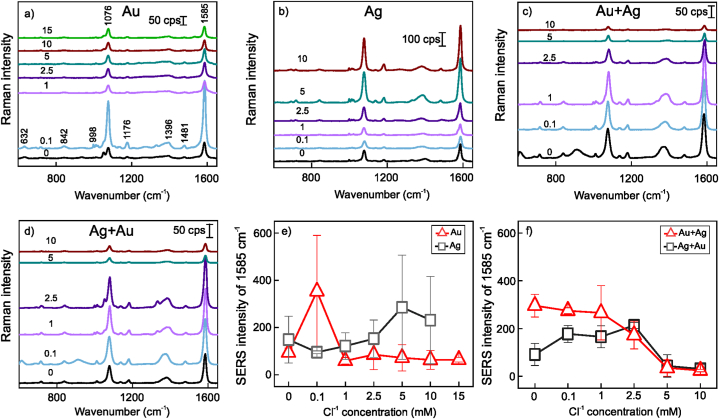
(a–d) SERS spectra of Au surface-adsorbed 4-MBA self-assembled monolayer obtained by using different nanoparticles (Au, Ag, Au + Ag, and Ag + Au). The Cl^−^ concentrations in ablation solutions are indicated above the respective spectra (mM). The laser excitation wavelength was 633 nm (45 μW). (e, f) SERS intensity dependence of 1585 cm^−1^ mode of 4-MBA monolayer on Cl^−^ concentration used in ablation liquid.

**Fig. 14:**
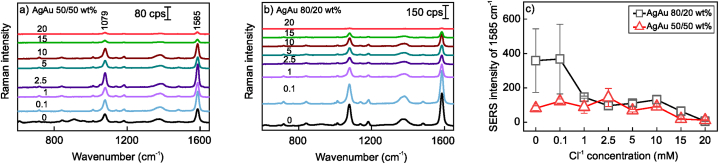
(a–b) SERS spectra of 4-MBA monolayer obtained using nanoparticles prepared laser ablation of AgAu 50/50 wt% and AgAu 80/20 wt% targets in saline aqueous solutions. The concentrations of Cl^−^ in mM are indicated above the respective spectra. The laser excitation wavelength was 633 nm (45 μW). (c) Dependence of 4-MBA SERS mode at 1585 cm^−1^ on the Cl^−^ concentration used in ablation liquid.

SERS sensitivity of monometallic, mixed, and alloy nanoparticles produced by laser ablation in saline water (KCl 0–20 mM) was probed using an Au surface-adsorbed 4-MBA self-assembled monolayer. Spectra in Fig. 13, Fig. 14 clearly show 4-MBA-related spectral modes at 1585 and 1076 cm^−1^ assigned to ring ν_8a_ motion and breathing vibration of the aromatic ring (ν_12_), respectively. 1350–1430 cm^−1^ spectral region contains broad features related to carboxylate group stretchings ν(COO^−^), whereas its deformation vibration δ(COO^−^) appears at 842 cm^−1^ [74]. Narrow and very weak spectral modes near 998 and 1020 cm^−1^ could be assigned to a small portion of the 4-MBA molecules that underwent decarboxylation during Au surface adsorption, which resulted in the formation of the thiophenol molecule [75]. The intensity of the dominant band near 1585 cm^−1^ was used to evaluate SERS enhancement in Fig. 13 e and f. Almost in all cases, SERS signal decrease was observed at the higher end of the KCl concentration range, which is related to fast nanoparticle agglomeration. Several compositions show higher variability in signal intensity, particularly Au 0.1 mM, Ag 5 mM, and Ag 10 mM, as well as AgAu 80/20 wt% abated in 0 and 0.1 M KCl (Fig. 14c). These changes can be attributed to the formation of so-called hot spots, which are irregular metal surface locations populated with vertices, sharp edges, etc., that lead to plasmonic coupling and a remarkable boost in a local electric field. However, the reason behind these compositions giving such a distinct response remains elusive. One of the approaches to cope with this issue could be an introduction of a magnetic counterpart to a plasmonics nanoparticle that would allow more even nanomaterial distribution in the external magnetic field [76,77]. Monometallic mixed Au and Ag nanoparticle solutions exhibited more directional SERS sensitivity towards chloride concentration in Fig. 13f. While the Cl^−^ concentration change from 0 to 0.1 mM doubled Ag + Au sensitivity, it had little effect on Au + Ag. Concentrations above 2.5 mM led to a sharp signal loss for both mixed compositions, while the range from 0 to 1 mM was optimal for Au + Ag, and the range from 0.1 to 2.5 mM for Ag + Au (Fig. 13f). The highest sensitivity was registered for alloy nanoparticles for 0- and 0.1-mM chloride solution ablated AgAu 80/20 wt% target (Fig. 14). Similarly to single-component nanoparticles, higher chloride concentrations led to a drastic decrease in spectral intensity.

Overall, the addition of low Cl^−^ concentrations (0.1 M) either increased the SERS intensity of the 4-MBA reporter molecule or had little effect, while the higher concentrations, depending on a target ablated, from 5 to 15 mM - had a diminishing effect on spectra. The SERS enhancement factor (EF) for the selection of nanoparticle cluster was calculated from the data in Fig. 13, Fig. 14 according to the previously described method [78,79]. The EFs were rather similar and ranged from 2 × 10^7^ to 3 × 10^8^, with the highest ones detected for Au at 0.1 mM and AgAu 80/20 wt% at 0.0 and 0.1 M chloride (Table 1). The EF is approximately similar to demonstrated in our previous work, in which similar nanoparticles were attached with magnetite to endow them with magnetic properties [80].

**Table 1:** SERS enhancement factors for different nanoparticle compositions obtained from 4-MBA self-assembled monolayer.

[Cl^−^], mM	Au	Ag	Ag + Au	Au + Ag	AgAu 50/50 wt%	AgAu 80/20 wt%
0	7.5 × 10^7^	1.2 × 10^8^	7.3 × 10^7^	2.4 × 10^8^	6.6 × 10^7^	2.9 × 10^8^
0.1	2.8 × 10^8^	7.6 × 10^7^	1.4 × 10^8^	2.2 × 10^8^	9.8 × 10^7^	2.9 × 10^8^
10	5.1 × 10^7^	1.9 × 10^8^	2.5 × 10^7^	1.9 × 10^7^	7.3 × 10^7^	1.1 × 10^8^

**Fig. 15:**
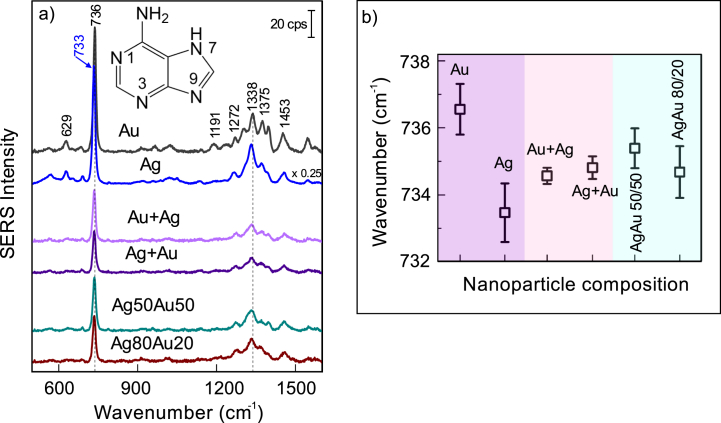
(a) SERS spectra of 10^−5^ M adenine obtained using different nanoparticles prepared in 0.1 mM KCl solution. The excitation wavelength was 633 nm (45 μW). Measurements from 15 spots on the surfaces were averaged. Inset shows the N7H tautomeric form of adenine. (b) The dependency of wavenumbers of adenine ring breathing mode on nanoparticle composition.

The adsorption of biological molecules on nanostructured Au and Ag surfaces is essential for medical and biological diagnostics and technological sectors. Thus, we have used the nanoparticles ablated 0.1 mM KCl solution to probe the adsorption of biologically relevant adenine molecules (Fig. 15). Adenine may form several adsorption complexes depending on its tautomerism and ionic form. The characteristic SERS signature of adenine is the highly intense band near 735 cm^−1^, which is attributed to the ring breathing mode [[81], [82], [83]]. The Raman spectrum of solid adenine exhibits this band at a considerably lower wavenumber, 723 cm^−1^ [83]. The mode is sensitive to the interaction of adenine with a surface, and accordingly, the DFT calculations predict interactions with Au and Ag surfaces to occur in N7H tautomeric form (see insert in Fig. 15a) [81,82]. The possible interaction sites are N3 and N9 atoms (for molecular structure see insert in Fig. 15a). A distinct separation in wavenumbers δ_Au-Ag_ = 3.1 cm^−1^ can be utilized in predicting the metallic substrate to which adenine molecules are adsorbed (Fig. 15b). Interestingly, when mixed monometallic nanoparticles are used, the dominant adenine mode position lands between those found for Au and Ag nanoparticles. Upon closer inspection, no significant broadening of the 735 cm^−1^ spectral mode was found for mixed and alloy nanoparticles. The full width at half maximum (FWHM) for Au and Ag was 5.9–6.4 cm^−1^, for Au + Ag and Ag + Au, 6.4 cm^−1^, and for AgAu alloy nanoparticles, 6.2–6.8 cm^−1^. This indicates the modification of surface properties and the presence of a single modified adsorption site, rather than two Au- and Ag-related surface sites, which would result in spectral overlap and broadening of the mode.

**Fig. 16:**
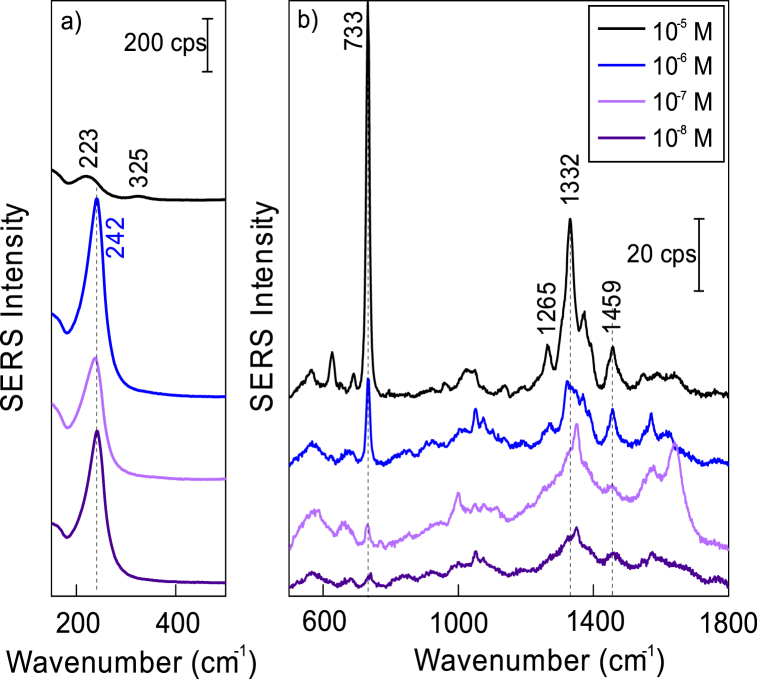
SERS spectra of 10^−5^–10^−8^ M adenine and bare Ag nanoparticles prepared in 0.1 mM KCl solution. The excitation wavelength was 633 nm (45 μW). Measurements from 15 spots on the surfaces were averaged.

Fig. 16 shows concentration-dependent SERS spectra of adsorbed adenine for bare Ag nanoparticles prepared in 0.1 mM KCl solution. The characteristic feature for surface-bound adenine became visible at 734 cm^−1^ in the SERS spectrum observed at solution concentration as low as 10^−8^ M (Fig. 16b). FWHM of this band was found to be relatively high, 20.1 cm^−1^. An increase in solution concentration results in an augment of intensity and substantial narrowing of the band. Thus, FWHM decreased to 11.9 cm^−1^ at a concentration of 10^−5^ M. In the low-frequency spectrum, the intense and broadband is visible near 242 cm^−1^ at adenine concentrations from 10^−8^ to 10^−6^ M (Fig. 16a). This band corresponds to the stretching vibration ν(Ag–Cl) of adsorbed chloride anions [[71], [72], [73],^84^,^85^]. The anions adsorb on the surface of nanoparticles in the synthesis stage; no chloride anions were present in the adenine adsorption solution. Direct observation of adsorbed chloride ions by SERS confirms the role of anions in the preservation of negative charge at the interface and stabilization of nanoparticles. Fig. 16 shows that in the adenine solution concentration region from 10^−8^ to 10^−6^ M adenine coadsorbs with Cl^−^ anions at the interface. However, at higher concentrations (10^−5^ M) adenine molecules replace anions from the surface as evidenced by the disappearance of an intense band near 242 cm^−1^ and the appearance of a new lower-intensity band at 223 cm^−1^. This band is associated with metal-adsorbate vibrational mode and reflects the direct bonding of adenine with nanoparticle surface [83].

The limit of detection (LOD) for adenine and Ag nanoparticles prepared in 0.1 mM KCl solution was found to be 10^−8^ M. Similar LOD were found for Au + Ag and AuAg nanoparticles in 0.1 mM KCl solutions. However, other preparations exhibited lower SERS sensitivity. In literature, SERS detection limits for adenine were observed in the range from 10^−6^ to 10^−14^ M [80]. The highest LOD values were obtained for graphene oxide coated with Ag NPs (10^−14^ [86]), cluster of Ag nanoparticles (10^−11^ [87]), Ag nanocubes coated with SiO_2_ (8.9 × 10^−10^ [88]), and Au NPs embedded in polymer matrix on graphene (10^−10^ [89]).

## 4 Conclusions

The colloidal solutions of gold, silver, mixed Au + Ag, Ag + Au, and alloys of AgAu 50/50 wt%, AgAu 80/20 wt% nanoparticles were generated to study their stability and SERS signal strength at different KCl salt concentrations in water. The particles produced in distilled water exhibited lower concentration, larger size, greater dispersion, and a darker color compared to the particles generated in the salt solution. In contrast, the particles in the salt solution were smaller in size, clearer in shape, and had less inter-particle adhesion. The optimal concentration of KCl salt has been determined to maintain colloid stability and decrease spectral deviations over 8 weeks. Furthermore, various KCl ranges have been identified as appropriate for different metal nanoparticles and their hybrids: Au 0.1–15 mM; Ag 0–2.5 mM; Au + Ag 0–10 mM; Ag + Au 0.1–10 mM; AgAu 50/50 wt% and AgAu 80/20 wt% 0.1–10 mM. Higher concentrations of KCl decrease the stability duration of colloidal solutions. All stable nanoparticles demonstrated a negative charge and SERS sensitivity. It has been observed that the SERS sensitivity experiences a marked increase when employing Au nanoparticles generated in a 0.1 mM KCl solution, and Ag nanoparticles created in a 5 mM KCl solution. Monometallic mixed solutions of Au and Ag nanoparticles revealed different reactions to chloride concentration. The sensitivity of Ag + Au doubled when the chloride concentration increased from 0 to 0.1 mM. On the other hand, there was little sensitivity change in Au + Ag for the same range. Nevertheless, both mixed compositions experienced a sharp signal loss at concentrations exceeding 2.5 mM. This is the upper limit of the chloride concentration at which an effective increase of SERS signal occurs for the following nanoparticles. For the Au + Ag mixture, it was discovered that the most effective range is between 0 and 1 mM chloride. Conversely, the range between 0.1 and 2.5 mM chloride produced the best outcomes for the Ag + Au mixture. The highest sensitivity was registered for alloy nanoparticles ablated in 0- and 0.1 mM chloride solutions using an AgAu 80/20 wt% target. Furthermore, it is noteworthy that the 0.1 mM alloy nanoparticles not only demonstrated marked improvement in the SERS signal, but also remained stable throughout the monitoring period. In addition, silver nanoparticles ablated in 0.1 mM chloride solutions allowed the identification of biologically relevant adenine molecules at concentrations of 10^−8^ M.

## CRediT authorship contribution statement

**Vita Petrikaitė:** Writing – review & editing, Writing – original draft, Visualization, Validation, Investigation, Formal analysis. **Martynas Talaikis:** Writing – review & editing, Visualization, Investigation, Formal analysis. **Lina Mikoliūnaitė:** Writing – review & editing, Project administration, Investigation, Formal analysis. **Aikaterini-Maria Gkouzi:** Investigation. **Romualdas Trusovas:** Writing – review & editing, Investigation, Formal analysis. **Martynas Skapas:** Investigation, Formal analysis. **Gediminas Niaura:** Writing – review & editing, Supervision, Project administration, Investigation, Funding acquisition, Formal analysis, Conceptualization. **Evaldas Stankevičius:** Writing – review & editing, Visualization, Supervision, Investigation, Formal analysis, Conceptualization.

## Declaration of competing interest

The authors declare that they have no known competing financial interests or personal relationships that could have appeared to influence the work reported in this paper.
