# Supplementary material for: Stability and SERS signal strength of laser-generated gold, silver, and bimetallic nanoparticles at different KCl concentrations
Source: Heliyon. 2024 Jul 18;10(15):e34815. doi: 10.1016/j.heliyon.2024.e34815 (PMC11320324; doi:10.1016/j.heliyon.2024.e34815)
Supplement: Multimedia component 1 [file mmc1.docx]

Supporting Information

Stability and SERS signal strength of laser-generated gold, silver, and bimetallic nanoparticles at different KCl concentrations

Vita Petrikaitė, Martynas Talaikis, Lina Mikoliūnaitė, Aikaterini-Maria Gkouzi, Romualdas Trusovas, Martynas Skapas, Gediminas Niaura, Evaldas Stankevičius*

**Author information**

**Corresponding Author**

**Evaldas Stankevičius** - Department of Laser Technologies, Center for Physical Sciences and Technology (FTMC), Savanoriu Av. 231, LT-02300, Vilnius, Lithuania;

orcid.org/0000-0003-3783-5506; Email: evaldas.stankevicius@ftmc.lt

**Authors**

**Vita Petrikaitė** - Department of Laser Technologies, Center for Physical Sciences and Technology (FTMC), Savanoriu Av. 231, LT-02300, Vilnius, Lithuania; Email: vita.petrikaite@ftmc.lt

**Martynas Talaikis** - Department of Organic Chemistry, Center for Physical Sciences and Technology (FTMC), Sauletekio av. 3, LT-10257, Vilnius, Lithuania; Email: martynas.talaikis@ftmc.lt

**Lina Mikoliūnaitė** - Department of Organic Chemistry, Center for Physical Sciences and Technology (FTMC), Sauletekio av. 3, LT-10257, Vilnius, Lithuania; Email: lina.mikoliunaite@ftmc.lt

**Aikaterini-Maria Gkouzi** - Department of Organic Chemistry, Center for Physical Sciences and Technology (FTMC), Sauletekio av. 3, LT-10257, Vilnius, Lithuania; Email: aikaterini.gkouzi@ftmc.lt

**Romualdas Trusovas** - Department of Laser Technologies, Center for Physical Sciences and Technology (FTMC), Savanoriu Av. 231, LT-02300, Vilnius, Lithuania; Email: romualdas.trusovas@ftmc.lt

**Martynas Skapas** - Department of Characterisation of Materials Structure, Center for Physical Sciences and Technology (FTMC), Sauletekio Av. 3, LT-10257, Vilnius, Lithuania;

Email: martynas.skapas@ftmc.lt

**Gediminas Niaura** - Department of Organic Chemistry, Center for Physical Sciences and Technology (FTMC), Sauletekio av. 3, LT-10257, Vilnius, Lithuania;

Email: gediminas.niaura@ftmc.lt

**Table S1**. The average size of each metal nanoparticle in different KCl solutions.

| Material | 0 mM | 0.1 mM | 1 mM | 2.5 mM | 5 mM | 10 mM | 15 mM | 20 mM |
| --- | --- | --- | --- | --- | --- | --- | --- | --- |
| Au | 24±18 nm | 7±3 nm | 20±5 nm | 19±4 nm | 8±5 nm | 11±4 nm | 13±5 nm | 28±12 nm |
| Ag | 33±14 nm | 5±2 nm | 6±2 nm | 10±4 nm | 18±9 nm | 8±5 nm | 16±8 nm | 19±9 nm |
| Au+Ag | 37±18 nm | 6±3 nm | 7±3 nm | 17±4 nm | 10±5 nm | 7±5 nm | 15±4 nm | 20±7 nm |
| Ag+Au | 35±30 nm | 12±6 nm | 12±7 nm | 12±5 nm | 9±4 nm | 16±6 nm | 14±7 nm | 24±15 nm |
| AgAu50/50 | 21±6 nm | 20±6 nm | 18±6 nm | 13±3 nm | 16±7 nm | 18±6 nm | 16±9 nm | 18±7 nm |
| AgAu80/20 | 43±19 nm | 17±5 nm | 13±3 nm | 19±6 nm | 13±6 nm | 15±4 nm | 13±5 nm | 22±13 nm |

**Table S2**. Concentrations of each metal nanoparticle in different KCl solutions.

| KCl /mM | 0 | 0,1 | 1 | 2,5 | 5 | 10 | 15 | 20 |
| --- | --- | --- | --- | --- | --- | --- | --- | --- |
| Au, mg/l | 90 | 95 | 115 | 180 | 135 | 135 | 135 | 105 |
| Ag, mg/l | 35 | 35 | 30 | 35 | 35 | 30 | 30 | 35 |
| Ag+Au, mg/l | 85 | 90 | 95 | 90 | 80 | 85 | 95 | 90 |
| Au+Ag, mg/l | 85 | 90 | 85 | 100 | 90 | 95 | 85 | 105 |

**Table S3.** Concentrations of Au and Ag nanoparticles in monometallic mixed solution at different concentrations of KCl solutions.

| Ag+Au, when first ablated silver, afterwards gold | | | | | | | | |
| --- | --- | --- | --- | --- | --- | --- | --- | --- |
| KCl, mM | 0 | 0,1 | 1 | 2,5 | 5 | 10 | 15 | 20 |
| Au NP concentration, mg/l | 60 | 65 | 75 | 75 | 70 | 70 | 75 | 80 |
| Ag NP concentration, mg/l | 25 | 25 | 20 | 15 | 10 | 15 | 20 | 10 |
| Au+Ag, when first ablated gold, afterwards silver | | | | | | | | |
| KCl, mM | 0 | 0,1 | 1 | 2,5 | 5 | 10 | 15 | 20 |
| Au NP concentration, mg/l | 65 | 60 | 55 | 75 | 80 | 75 | 70 | 85 |
| Ag NP concentration, mg/l | 20 | 30 | 30 | 25 | 10 | 20 | 15 | 20 |

**Table S4**. Zeta potential of the stable colloidal solutions.

| Cl^-1^ concentration (mM) | Au | Ag | Au+Ag | Ag+Au | Ag50Au50 | Ag80Au20 |
| --- | --- | --- | --- | --- | --- | --- |
| 0 | -14 | -43.58 | -51.37 | - | - | - |
| 0.1 | -53.16 | -47.93 | -54.43 | -51.52 | -57.26 | -43.92 |
| 1 | -51.84 | -52.5 | -56 | -51.95 | -54.23 | - |
| 2.5 | -55 | - | -41.15 | -45.65 | -57.98 | -55.55 |
| 5 | -19 | - | -48.29 | -49.4 | -55.37 | -58.79 |
| 10 | -17 | -18 | -29.34 | -16.32 | -44.03 | -44.83 |
| 15 | - | - | - | - | - | - |
| 20 | - | - | - | - | - | - |
